# Insulin Receptor Substrate 1 Gene and Glucose Metabolism Characteristics in Type 2 Diabetes Mellitus with Comorbidities

**DOI:** 10.4314/ejhs.v31i5.12

**Published:** 2021-09

**Authors:** Mariya Marushchak, Inna Krynytska

**Affiliations:** 1 Department of Functional and Laboratory Diagnostics, I. Horbachevsky Ternopil National Medical University, Ternopil, Ukraine; 2 Department of Functional and Laboratory Diagnostics, I. Horbachevsky Ternopil National Medical University, Maydan Voli, 1, 46001 Ternopil, Ukraine

**Keywords:** Type 2 diabetes mellitus, comorbidities, insulin resistance, IRS1 gene

## Abstract

**Background:**

Genetic variants that affect insulin signaling play an important role in insulin resistance (IR) in type 2 diabetes mellitus (T2DM). This study aimed to evaluate changes of the glycemic profile and IR in T2DM with comorbid obesity and chronic pancreatitis (CP) considering the allele status of the IRS1 gene (rs2943640).

**Methods:**

The study involved 33 type-2 diabetic patients and 10 healthy individuals. The IRS-1 gene rs2943640 C>A polymorphism was genotyped using the TaqMan real-time PCR method.

**Results:**

In type 2 diabetic patients regardless of the presence/absence of comorbid obesity and CP glycemic profile parameters significantly did not differ between carriers of allele C or allele A of the IRS1 gene (rs2943640). At the same time significantly higher HOMA-IR (by 2.25 times) was established in carriers of the C allele. In type 2 diabetic patients with both comorbidities (carriers of the C allele) the maximum HOMA-IR was established, which significantly differed from the data of patients with only T2DM and patients with comorbid obesity. In carriers of the A allele significantly higher level of HOMA-IR was found in patients with comorbid obesity and CP vs patients with only T2DM, and also in patients with comorbid obesity vs patients with only T2DM.

**Conclusions:**

Presence of the C allele of the IRS1 gene (rs2943640) may indicate risk of high IR in type 2 diabetic patients regardless of the presence/absence of comorbid obesity and CP; here with CP is a more important factor in IR progression then obesity.

## Introduction

Diabetes Mellitus (DM) is among the most widespread endocrine disorders; if left untreated it, it leads to a variety of complications which can be life-threatening (1–4). A report from the International Diabetes Federation shows that in 2019 463 million people were diagnosed with DM worldwide, and of this figure, the majority (91%) suffered from type 2 diabetes (T2DM). It is projected that by 2045, the incidence of DM might reach 700 million, or at least 10% of the global population (5). Among the most severe T2DM complications associated with increased morbidity and mortality are disorders of micro- and macrovascular systems (6). These effects contribute to a substantial economic and healthcare burden: worldwide, the cost of DM treatment in 2019 exceeded 727 billion dollars (5). Therefore, it is vital to explain the molecular mechanisms underlying the development and progression of T2DM (7).

The presence of other comorbidities or chronic diseases drastically affects DM patient outcomes, treatment and management options, and associated healthcare expenses (8). While the hallmark of T2DM is insulin resistance (IR), it is associated with other metabolic disorders such as dyslipidemia and obesity (9, 10). This is because excess abdominal fat and concomitant dysfunction and inflammation of the adipose tissue increase secretion of adipocytokines, which produce diabetogenic effect. In particular, this group of cytokines can disrupt insulin action in skeletal muscle, liver, brain, and other organs (11). Another disorder associated with obesity, IR and T2DM is chronic pancreatitis (CP) (12). Zhuravleva L.V. and Shekhovtsova Y.A. suggested that exocrine insufficiency of the pancreas was diagnosed in 53.3% of T2DM patients (13).

IR is the most common metabolic disorder associated with T2DM. While environmental factors, such as diet and physical activity have a substantial effect on IR, genetic variants that affect insulin signaling play an important role. Among the genes involved in insulin signaling pathways, is the insulin receptor substrate 1 (*IRS-1*) which encodes IRS-1 protein involved in transmitting the signal between the insulin receptor and phosphoinositide 3-kinase (PI3K) (14). As IRS-1 phosphorylated on insulin receptor, it binds other proteins, which results in increase of the glucose transporter type 4 (GLUT4) molecules on the outer membrane of muscle and adipose tissues. This in turn intensifies glucose uptake into these tissues from the blood. Dysregulation of IRS-1 expression and function was shown to disrupt the insulin signaling pathway and can produce IR and DM (15,16). In several populations, *IRS-1* polymorphisms have been associated with IR and T2DM (17, 18, 19). However, a study of the effects of IRS-1 polymorphisms Gly972Arg and Ala513Pro in a non-obese Turkish population did not find a link between them and risk of developing T2DM and its phenotypes (20).

Thus, mutations and variants of *IRS1* may be of interest for clinical researchers as factors potentially affecting development and progression of metabolic diseases, such as T2DM. Moreover, there have been no previous studies of *IRS1* gene polymorphisms among T2DM patients in Ukraine, be it with only T2DM, or comorbid with obesity or CP. Therefore, the aim of our study was to evaluate changes of the glycemic profile parameters and insulin resistance in type 2 diabetic patients with comorbid obesity and chronic pancreatitis considering the allele status of the *IRS1* polymorphism rs2943640.

## Materials and Methods

**Characteristics of the participants**: The study involved 33 type-2 diabetic patients hospitalized to the Endocrinology Department of Ternopil University Hospital (Ternopil, Ukraine) in 2019–2020 and 10 healthy individuals. The distribution of individuals to study groups is presented in Table 1.

**Table 1:** Characteristics of the study groups (n=43)

Group	Patient cohort	n	%
1	T2DM patients with normal body weight without CP	9	20.8
2	T2DM patients with overweight/obesity without CP	14	32.6
3	T2DM patients with overweight/obesity and CP	10	23.3
4	Healthy individuals (control)	10	23.3

There were no significant age and sex differences between the groups in this study. The frequency distribution of rs2943640 genotypes and the verification of conformity with Hardy-Weinberg equilibrium for the population were performed in the study and control groups. It was found that the frequencies of the C/A genotype of *IRS-1* rs2943640 in type 2 diabetic patients without comorbidities and with comorbid obesity and CP did not significantly deviate from Hardy-Weinberg equilibrium (p>0.05) (Table 2).

**Table 2:** IRS-1 rs2943640 genotypes in study groups and their conformity to Hardy-Weinberg equilibrium

Genotypes		T2DM (Group 1)		T2DM + obesity (Group 2)		T2DM + obesity + CP (Group 3)	
		
		Expected	Present	Expected	Present	Expected	Present
Homozygotes that are common	C/C	1	0	4.0	3	6.4	7
Heterozygotes	C/A	4	6	7.0	9	3.2	1
Homozygotes that are rare	A/A	4	3	3.0	2	0.4	2
χ^2^, p		χ^2^=2.25; p>0.05		χ^2^=1.20; p>0.05		χ^2^=2.45; p>0.05	

Note: * - statistically significant results

Inclusion criteria: clinical, laboratory and instrumental signs of T2DM, CP and obesity, no sharp increase (exceeding normal activity not more than 3 times) of blood serum alpha-amylase, lipase, alanine aminotransferase, aspartate aminotransferase, alkaline phosphatase and gamma-glutamyl transferase.

Exclusion criteria from the study: signs of clinically significant neurological, mental, renal, hepatic, immune, gastrointestinal, urogenital disorder; injuries of the musculoskeletal system, skin, sense organs, endocrine system (except T2DM); or uncontrolled hematologic diseases; acute pancreatitis, unstable or life-threatening heart disease; patients with malignant neoplasms who have not been in complete remission for at least 5 years, medication (drug) dependence, and alcohol dependence.

T2DM diagnoses were confirmed according to the 2019 Recommendations of the American Diabetes Association (ADA) (21). The diagnosis criteria use the level of glycated haemoglobin (HbA1c) (≥6.5%), which was determined using an automatic biochemical analyzer COBAS 6000 (Roche Hitachi, Germany) and plasma glucose level, which was determined on an automatic biochemical analyzer BAS INTEGRA® 400 (Roche Diagnostics) using a standard set. Plasma insulin was determined by the help of enzyme-linked immunosorbent analyzer “Thermo Scientific Multiskan FC” using DRG set (Germany). HOMA-IR (Homeostasis Model Assessment for Insulin Resistance) index was used to determine IR. It was calculated using the formula: HOMA-IR=(fasting plasma glucose, mmol/l × fasting plasma insulin, µIU/ml)/22.5 (22). CP diagnoses were following the recommendations of the American Pancreatic Association (23). Body mass index (BMI) was calculated using the formula: body weight (kg)/height (m^2^). Data were interpreted according to the WHO guidelines: normal weight in the range of 20.0–24.9 kg/m^2^; overweight (pre-obesity), 25.0–29.9 kg/m^2^; class 1 obesity, 30.0–34.9 kg/m^2^; class 2 obesity, 35.0–39.9 kg/m^2^ and class 3 obesity, > 40 kg/m^2^.

**Sample preparation**: Genomic DNA was extracted from peripheral blood leukocytes using a commercially available DNA isolation kit (QIAamp Blood DNA Mini Kit, QIAGEN, Germany).

**Genetic analyses**: The *IRS-1* gene rs2943640 C>A polymorphism was genotyped using the TaqMan real-time PCR method (Applied Biosystems, Foster City, CA, USA) (24). Quality control was performed with eight negative control and positive control samples in each 96-well plate. In addition, approximately 10% of the samples were randomly selected for further quality control, and the concordance rate was 100%. Amplification of the 25-bp *IRS-1* sequence including rs2943640 was performed by using PCR with 5′-GAAATGAGAGGAACCCTTCTAACTA-3′ as the forward primer and 5′-AGGAACTCTTCTAACTATTAGCCC-3′ as the reverse primer. Two alleles of the rs2943640 *IRS-1* polymorphism were detected (C and A).

**Ethics**: The ethical principles included in the Declaration of Human Rights adopted in Helsinki, in 1975, and revised in 2008, were fully respected in our study. The enrolled subjects participated in this study voluntarily, completed and signed a written informed consent. Study protocol was approved by the Ethics Committee of I. Horbachevsky Ternopil National Medical University.

**Statistics**: Study results were analyzed using STATISTICA 7.0 and MedCalc software. The Kolmogorov-Smirnov test was used to compare probability distributions. Quantitative values, due to their non-parametric distribution, are presented in the form of median, lower, and upper quartiles, and compared using the Mann-Whitney test. For frequency values, the percentage ratio and its 95% confidence interval were calculated, and their comparative analysis was performed using Pearson's chi-square test and Fisher's bilateral test, its 95% confidence interval (CI) and significance coefficient p-value were determined.

## Results

Table 3 presents the indices of glucose metabolism in control group and type 2 diabetic patients considering the presence/absence of comorbid obesity or CP. When conducting the analysis in the Kruskal-Wallis test by ranks it was found that the studied indices significantly differed between groups of patients with only T2DM, T2DM+obesity and T2DM+obesity+CP. It should be noted that the highest rates of glycemic profile parameters (HbA_1_c) and IR (level of insulin and HOMA-IR) were established in type 2 diabetic patients with comorbid obesity and CP (Table 3).

**Table 3:** The indices of glucose metabolism in type 2 diabetic patients taking into account the presence/absence of comorbid obesity or CP

Group of the Patients	Insulin, µIU/ml	Glucose, mmol/l	HOMA-IR	HbA_1_c, %
Only T2DM (n=9)	19.27 (18.16; 21.25)	7.12 (6.28; 7.60)	5.97 (5.73; 7.11)	6.80 (6.30; 7.50)
T2DM + Obesity (n=14)	48.69 (45.18; 51.23)	7.84 (7.25; 9.14)	16.63 (14.14; 19.99)	7.30 (6.60; 7.80)
T2DM + Obesity + CP(n=10)	80.84 (79.23; 81.80)	7.80 (7.31; 7.87)	27.71 (25.94; 28.36)	9.90 (7.70; 10.00)
Control (n=10)	10.82 (10.24; 11.24)	3.80 (3.80; 4.10)	1.78 (1.71; 2.10)	4.30 (4.20; 4.80)
Kruskal-Wallis criterion	H=39.13;	H=23.06;	H=37.84;	H=26.53;
	p<0.001*	p<0.001*	p<0.001*	p<0.001*

Where: * - statistically significant results

In multiple comparison the indices of glucose metabolism their values were significantly higher in studied groups vs control (p<0.022–0.001). Level of insulin and HOMA-IR were significantly higher in patients with T2DM+obesity+CP vs patients with only T2DM, respectively by 4.20 and 4.64 times (p<0.001).

Table 4 presents the glucose metabolism abnormalities in patients with T2DM regardless of the presence/absence of comorbid obesity or CP, taking into account the allele status of the *IRS1* gene (rs2943640). It was found that glycemic profile parameters significantly did not differed between carriers of Allele C or Allele A. At the same time significantly, higher indices of IR were established in carriers of the C allele (level of insulin - by 2.20 times and HOMA-IR - by 2.25 times) vs carriers of the A allele (Table 4).

**Table 4:** The indices of glucose metabolism in type 2 diabetic patients regardless of the presence/absence of comorbid obesity or CP taking into account the allele status of the *IRS-1* gene (rs2943640)

Index	Allele C (n=47)	Allele A (n=39)	p
Insulin, µIU/ml	48.72 (17.69; 79.23)	22.14 (11.67; 48.72)	0.046*
Glucose, mmol/l	7.31 (5.67; 7.87)	7.12 (4.50; 7.87)	0.482
HOMA-IR	16.02 (5.75; 25.94)	7.11 (2.30; 18.76)	0.048*
HbA_1_c, %	7.30 (6.00; 7.80)	6.60 (5.20; 7.50)	0.208

Where: * – statistically significant results

Analysis of the effect of *IRS1* gene polymorphism (rs2943640) on glucose metabolism abnormalities in the Kruskal-Wallis test by ranks found that level of glucose and HbA_1_c significantly differed within the studied and control groups both in carriers of the C allele and in carriers of A allele (table 5). It should be noted that studied glycemic profile parameters in patients with only T2DM and in patients with T2DM+obesity and T2DM+obesity+CP significantly did not differ from each other in carriers of the C allele and in carriers of A allele *IRS1* gene (rs2943640). Regarding indices of IR, the level of insulin and HOMA-IR in patients with T2DM+obesity and T2DM+obesity+CP were significantly higher vs control, whereas in patients with only T2DM no significant difference with control was found (Table 5).

**Table 5:** Analysis of the effect of *IRS1* gene polymorphism (rs2943640) on glucose metabolism abnormalities in type 2 diabetic patients with comorbid obesity and CP considering the allele status

Group of the Patients		Insulin, µIU/ml	Glucose, mmol/l	HOMA-IR	HbA_1_c, %
Only T2DM (1)	Allele C (n=6)	18.72^3^ (17.69;21.25)	7.60(7.12;8.30)	6.54^3^ (5.75; 7.18)	7.40 (6.30; 7.50)
	Allele A (n=12)	19.91^2^,^3^(18.35;21.70)	6.70 (6,28; 7.60)	5.86^3^ (5,67; 7.06)	6.65 (6.30; 7.40)
T2DM + Obesity	Allele C (n=15)	48.72^3^ (44.56; 51.23)	7.80 (7.25; 9.23)	16.02^3^(14.14;19.99)	7.30 (6.80; 7.80)
(2)	Allele A (n=13)	48.66^1^ (46.94; 51.23)	8.68 (7.25; 9.14)	18.76^1^(14.14; 19.99)	7.20 (6.60; 7.60)
T2DM +	Allele C (n=16)	80.45^1^,^2^(78.87;81.86)	7.59 (6.66; 9.20)	27,11^1^,^2^(23.83;31.77)	8.85(6.85; 10.35)
Obesity+CP (3)	Allele A (n=4)	81.22^1^ (80.64; 81.80)	7.80 (7.80; 7.80)	28.16^1^(27,96; 28.36)	9.90 (9.90; 9.90)
Control	Allele C (n=10)	10.82^2^,^3^(10.24;11.24)	3.80^1^,^2^,^3^(3.80;4.10)	1.78^2^,^3^ (1.71; 2.10)	4.30^1^,^2^,^3^(4.20;4.80)
	Allele A (n=10)	10.82^2^,^3^(10.24;11.24)	3.80^1^,^2^,^3^(3.80;4.10)	1.78^2^,^3^ (1.71; 2.10)	4.30^1^,^2^,^3^(4.20;4.80)
H; p for Allele C		H=42.20;p<0.001	H=23.28;p<0.001	H=40.81; p<0.001	H=26.14; p<0.001
H; p for Allele A		H=34.86; p<0.001	H=23.67; p<0.001	H=33.92; p<0.001	H=27.81; p<0.001

Where: # – confidence level p<0.05 when comparing the values of indices in case of C or A allele presence within one study group; H; p – Kruskal-Wallis test and its confidence level

1 confidence level p<0.01 when comparing the values of indices in case of C or A allele presence within one study group vs patients with only T2DM

2 confidence level p<0.01 when comparing the values of indices in case of C or A allele presence within one study group vs patients with T2DM + Obesity

3 confidence level p<0.01 when comparing the values of indices in case of C or A allele presence within one study group vs patients with T2DM + Obesity + CP

In carriers of the C allele of the *IRS1* gene (rs2943640) - patients with T2DM+obesity+CP maximum insulin level and HOMA-IR were found, which significantly differed from the data of patients with only T2DM (respectively by 329.75 % and 65.13 %) and from the data of patients with T2DM+obesity (respectively by 314.53 % and 69.23 %) (Table 5).

In carriers of the A allele of the *IRS1* gene (rs2943640) significantly higher level of insulin (by 4.08 times) and HOMA-IR (by 4.81 times) were found in patients with T2DM+obesity+CP vs patients with only T2DM, and also in patients with T2DM+obesity higher level of insulin (by 2.44 times) and HOMA-IR (by 3.20 times) were found vs patients with only T2DM. At the same in patients with T2DM+obesity the level of plasma insulin and HOMA-IR significantly did not differ from the data of the patients with T2DM+obesity+CP (Table 5).

## Discussion

IR underpins both obesity and T2DM, since it impairs cellular response to insulin affecting carbohydrate, lipid, and protein metabolism, and resulting in high blood glucose levels. In adipose tissue, reduced cell sensitivity to insulin or impairment of the insulin pathway produce a wide range of effects on metabolic processes, making it is one of the most important hormones regulating anti-lipolytic processes (25). The insulin receptor substrate (IRS) proteins are signal adaptor proteins which play a role in signal transduction from both the insulin and IGF-1 receptors. IRS protein family includes four proteins, namely IRS-1, IRS-2, IRS-3, and IRS-4. IRS-1, identified the first, is encoded by a gene located on the chromosome 2q34-37. Notably, it is transcribed as a single exon (26). The phosphorylation of tyrosine residues on IRS-1 introduces binding sites for phosphoinositide 3-kinase (PI3K), which phosphorylates phosphatidylinositol 2 phosphate (PIP2) producing phosphatidylinositol 3 phosphate (PIP3), which in turn activates protein kinase B (Akt). Following this, Akt phosphorylates AMP-activated protein kinase (AMPK), which activates translocation GLUT4 containing vesicles to the plasma membrane, increasing glucose uptake. Thus, dephosphorylation of IRS-1 can potentially inhibit the entire insulin signal transduction pathway, resulting in IR (27).

In the present study we found that in type 2 diabetic patients irrespective of the presence/absence of comorbid obesity or/and CP glycemic profile parameters significantly did not differ between carriers of allele C or allele A of the *IRS1* gene (rs2943640). At the same time significantly higher (2.25 times) HOMA-IR was established in carriers of the C allele vs carriers of the A allele, indicating a more marked IR.

A positive correlation between *IRS-1* rs2943640 allele C and T2DM risk was found in the InterAct study involving 12,403 incident T2D cases and a representative cohort of 16,154 individuals of European descent (28). Rung J. et al. found an association between a rs2943641 C > T SNP, located ca. 500 kb downstream from the *IRS-1* locus, and T2DM risk, where the major C allele was associated with 19.0% risk increase (29). Importantly, this allele was also associated with reduced basal levels of IRS-1 protein and decreased insulin mediation of IRS-1 induced PI3K activity in human skeletal muscle. Interestingly, carriers of the rs2943641 C/C genotype had larger weight loss and improved IR than individuals without this genotype after being placed on a highcarbohydrate and low-fat diet, indicating a possible long-term effect this genotype on improving IR (14).

The potential mechanisms underlying the effect of these polymorphisms are unknown, but lipid-induced IR is likely to play a role (30). Chronic elevated free fatty acids (FFAs) in blood plasma impair insulin-associated glucose transport, uptake, and utilization in hepatocytes, adipocytes, and skeletal myocytes both in obesity as well as T2DM (31). Elevated FFAs levels also inhibit glycogen synthesis. Furthermore, long-term exposure to FFAs induces expression of inflammatory factors and activation of c-Jun N-terminal kinase (JNK), accelerating FA β-oxidation. This increases the rate of mitochondrial respiration, and resulting of surplus of electrons generates excess of reactive oxidant species (32). These factors can further impair insulin signaling molecules by lowering the phosphorylation levels of IRS and Akt, as well as expression and translocation of GLUT4, ultimately resulting in dysregulation of glucose uptake and utilization (33). Previous studies have demonstrated that extended high fat diets with elevated plasma FFAs levels impair insulin signaling by altering IRS-1 phosphorylation and reducing PI3K activation (34, 35).

Since we found the highest plasma insulin and HOMA-IR levels inpatients with T2DM+obesity+CP who were carriers of the C allele of the *IRS-1* rs2943640 compared to the patients with only T2DM and patients with T2DM+obesity, this suggests important contribution of CP to IR progression in this cohort of diabetic patients. CP is chronic inflammatory process, and as such, can contribute to IR emergence. For instance, 75% of patients with CP were reported to have developed IR, even in the absence of obesity (36). CP is characterized by the pancreatic inflammatory response with invasion of inflammatory cells, release of pro-inflammatory cytokines, and progressing fibrosis resulting in destruction of exocrine and endocrine cells (37, 38). Moreover, IR is associated with dysregulated production of pancreatic hormones, including insulin, glucagon and pancreatic polypeptide (39).

In this study, the carriers of rs2943640 A allele had significantly higher levels of plasma insulin and HOMA-IR in the groups of patients with T2DM+obesity+CP vs. patients with only T2DM, and also in patients with T2DM+obesity vs. patients with only T2DM. At the same time, levels of plasma insulin and HOMA-IR in the patients with T2DM+obesity did not significantly differ from that of the patients with T2DM+obesity+CP, suggesting important contribution of obesity to IR progression in T2DM in this cohort of patients. Chronic inflammation and hypoxia of adipose tissue are some of the effects of obesity, inducing abnormal production of cytokines, growth factors, FFAs and activating pro-inflammatory pathways. Up-regulation of the JNK and NF-κB pathways and inflammatory mediators can results in IR (40).

A strength of our study is that it is the first investigation of *IRS-1* gene polymorphisms among T2DM patients with comorbid obesity and CP in Ukrainian population. Identification of genes polymorphisms contributing to T2DM pathogenesis and treatment response is the first step towards the development of personalized medicine. Researching DM-linked gene polymorphisms can help to clarify heterogeneity of the disease and estimate its severity, which in turn will aid in developing an appropriate treatment corresponding to the patient's unique diabetes pathogenesis.

This study involved a small sample size; because of it, significant relationships between study factors were difficult to establish. The patients included in T2DM + obesity and T2DM + obesity + CP groups, were not randomly selected, potentially resulting in selection bias. While we cannot support the assumption that study participants represent the population of type 2 diabetic patients with comorbid obesity and CP in Ternopil region, but obtained results give grounds for further studies with larger sample sizes reflecting a more inclusive population.

Thus, the presence of the C allele of the *IRS1* gene (rs2943640) may indicate risk of high insulin resistance in type 2 diabetic patients regardless of the presence/absence of comorbid obesity and chronic pancreatitis; in type 2 diabetic patients (carriers of C allele of the *IRS1* gene (rs2943640)) with comorbid obesity and chronic pancreatitis, actually, chronic pancreatitis is more important factor in insulin resistance progression.
